# Complete mitochondrial genomes and updated divergence time of the two freshwater clupeids endemic to Lake Tanganyika (Africa) suggest intralacustrine speciation

**DOI:** 10.1186/s12862-022-02085-8

**Published:** 2022-11-03

**Authors:** Leona J. M. Milec, Maarten P. M. Vanhove, Fidel Muterezi Bukinga, Els L. R. De Keyzer, Vercus Lumami Kapepula, Pascal Mulungula Masilya, N’Sibula Mulimbwa, Catherine E. Wagner, Joost A. M. Raeymaekers

**Affiliations:** 1 Faculty of Biosciences and Aquaculture, Nord University, Universitetsalléen 11, 8026 Bodø, Norway; 2 Centre for Environmental Sciences, Research Group Zoology: Biodiversity and Toxicology, Hasselt University, Agoralaan Gebouw D, 3590 Diepenbeek, Belgium; 3 Laboratory of Biodiversity and Evolutionary Genomics, Department of Biology, KU Leuven, Charles Déberiotstraat 32, 3000 Leuven, Belgium; 4 Centre de Recherche en Hydrobiologie-Uvira (CRH-Uvira), Uvira, Sud-Kivu Democratic Republic of Congo; 5 Evolutionary Ecology Group (EVECO), Universiteit Antwerpen, Campus Drie Eiken, Universiteitsplein 1, 2610 Wilrijk, Belgium; 6 Université Catholique de Louvain, Place Sainte Barbe 2, 1348 Louvain-la-Neuve, Belgium; 7 Unité d’Enseignement et de Recherche en Hydrobiologie Appliquée (UERHA)-ISP/Bukavu, Bukavu, Sud-Kivu Democratic Republic of Congo; 8 University of Wyoming, 1000 E University Ave, Laramie, WY 82071 USA

**Keywords:** Great Lakes, Clupeiformes, Mitogenome, Time calibration, Phylogenetics

## Abstract

**Background:**

The hydrogeological history of Lake Tanganyika paints a complex image of several colonization and adaptive radiation events. The initial basin was formed around 9–12 million years ago (MYA) from the predecessor of the Malagarasi–Congo River and only 5–6 MYA, its sub-basins fused to produce the clear, deep waters of today. Next to the well-known radiations of cichlid fishes, the lake also harbours a modest clade of only two clupeid species, *Stolothrissa* *tanganicae* and *Limnothrissa* *miodon.* They are members of Pellonulini, a tribe of clupeid fishes that mostly occur in freshwater and that colonized West and Central-Africa during a period of high sea levels during the Cenozoic. There is no consensus on the phylogenetic relationships between members of Pellonulini and the timing of the colonization of Lake Tanganyika by clupeids.

**Results:**

We use short-read next generation sequencing of 10X Chromium libraries to sequence and assemble the full mitochondrial genomes of *S.* *tanganicae* and *L.* *miodon.* We then use Maximum likelihood and Bayesian inference to place them into the phylogeny of Pellonulini and other clupeiforms, taking advantage of all available full mitochondrial clupeiform genomes. We identify *Potamothrissa* *obtusirostris* as the closest living relative of the Tanganyika sardines and confirm paraphyly for *Microthrissa.* We estimate the divergence of the Tanganyika sardines around 3.64 MYA [95% CI: 0.99, 6.29], and from *P.* *obtusirostris* around 10.92 MYA [95% CI: 6.37–15.48].

**Conclusions:**

These estimates imply that the ancestor of the Tanganyika sardines diverged from a riverine ancestor and entered the proto-lake Tanganyika around the time of its formation from the Malagarasi–Congo River, and diverged into the two extant species at the onset of deep clearwater conditions. Our results prompt a more thorough examination of the relationships within Pellonulini, and the new mitochondrial genomes provide an important resource for the future study of this tribe*,* e.g. as a reference for species identification, genetic diversity, and macroevolutionary studies.

**Supplementary Information:**

The online version contains supplementary material available at 10.1186/s12862-022-02085-8.

## Background

Lake Tanganyika has experienced a turbulent geological history of lake level fluctuations, shifting shorelines and transient hydrological connections, paving the way for a complex sequence of colonisations that gave rise to a diverse freshwater fauna with a high degree of endemism [1]. The lake was originally formed by lateral expansion of the western branch of the East African rift, crossing the predecessor of the Malagarasi–Congo River around 9–12 million years ago (MYA). Only 5–6 MYA its water levels rose high enough for the sub-basins and the swampy areas in between to fuse into the deep clearwater lake of today [2–4]. The lake has experienced large water level fluctuations since then [5, 6]. These events are reflected in the evolutionary history of the organisms inhabiting the lake. For example, the adaptive radiations of the Tanganyika cichlid tribes happened over several stages, with some ancestral species colonizing the lake in the early stages of its formation, while others diversified later when the historical sub-basin lakes fused [7, 8] or following the depression of the northernmost sub-basin around 7–8 MYA [9]. Yet other lineages were initially thought to have colonized the lake at an even later stage, and thus established themselves in an already present adaptive radiation [10, 11]. Recent work, however, suggests that the cichlid radiation unfolded completely within the temporal and spatial confines of Tanganyika [9, 12–14].

Next to this textbook example of adaptive radiation, Lake Tanganyika also harbours a small clade of two endemic clupeid species, *Stolothrissa* *tanganicae* and *Limnothrissa* *miodon*. These clupeids are members of the African clupeid tribe Pellonulini, one of the most diverse freshwater radiations of Clupeiformes with 22 species in 11 genera, most occurring either on the West coast of Africa (distribution from Senegal down to Congo/Angola), or in the Congo River system and its tributaries and lakes [15, 16]. The members of Pellonulini are thought to be derived from a group of sardine-like species whose ancestors originated from the Atlantic West coast of Africa during a period of high sea levels between 30 and 50 MYA [16–18]. The exact route this radiation took through the Congo Basin is unknown, and the relationships between pellonuline taxa remain inconsistent in published clupeid phylogenies [17, 19–21].

The Tanganyika sardines are the fully pelagic, planktivorous, endemic *S.* *tanganicae* and the semi-pelagic, more opportunistic *L.* *miodon*, which is originally endemic to Lake Tanganyika but has also established in other lakes in Central Africa after anthropogenic introductions. Both species are important fisheries targets in Lake Tanganyika and provide food and livelihood for millions of people [22, 23]. The colonization and subsequent speciation of the Tanganyika sardines has only been explicitly addressed once [17], and estimated as part of larger phylogenies twice more [19, 20]. In these studies, estimates of their divergence time are based on minimum one and maximum three mitochondrial genes, and show substantial variation, the youngest being at 3.91 MYA and the oldest at 8 MYA with a large credibility interval (CI). Lake Tanganyika was formed 9–12 MYA, with the northern and southern sub-basins forming at 7–8 MYA and 2–4 MYA, respectively. The fusion of the sub-basins and onset of clearwater conditions is estimated at 5–6 MYA. Keeping these estimates in mind, a divergence time of the two sardine species of 8–10 MYA would mean that the lineage leading to *S.* *tanganicae* and *L.* *miodon* started to undergo speciation soon after entering the not yet connected sub-basins of the proto-lake. An older divergence time would indicate riverine speciation and subsequent colonization of the proto-lake. In contrast, a more recent divergence time would agree with intralacustrine speciation i.e. after the sub-basins of the lake connected to form the deep rift lake we see today.

Robust phylogenies and estimates of divergence time between lineages are crucial to understanding the relationship between geological or hydrological events, speciation and realised biodiversity. Mitochondrial genes are routinely used for this purpose [24], but single-gene datasets have limited ability to recover true phylogenetic relationships, especially in more closely related species [13, 25, 26] and tend to overestimate divergence time [27]. Whole mitochondrial genomes can contain phylogenetic information that is lost when targeting a single gene, and have yielded higher resolution and better supported phylogenies in recent studies of fish [28, 29] and other vertebrates [27, 30, 31], especially when investigating recently diverged or taxonomically diverse taxa [27].

In this study, we use short-read next generation sequencing (NGS) to sequence and assemble the complete mitochondrial genomes of *S.* *tanganicae* and *L.* *miodon.* We then use the new sequences, together with all available full mitochondrial clupeiform genomes, to build the first phylogeny of members of Pellonulinae to include all mitochondrial protein-coding genes (PCGs), rRNA-genes and the D-loop (control region). We revisit the phylogenetic relationships within Pellonulinae and estimate the divergence time of the Lake Tanganyika sardines with improved resolution. We discuss the results in the light of the geological history of Lake Tanganyika.

## Results

### New mitochondrial genomes, diversity and divergence

**Fig. 1:**
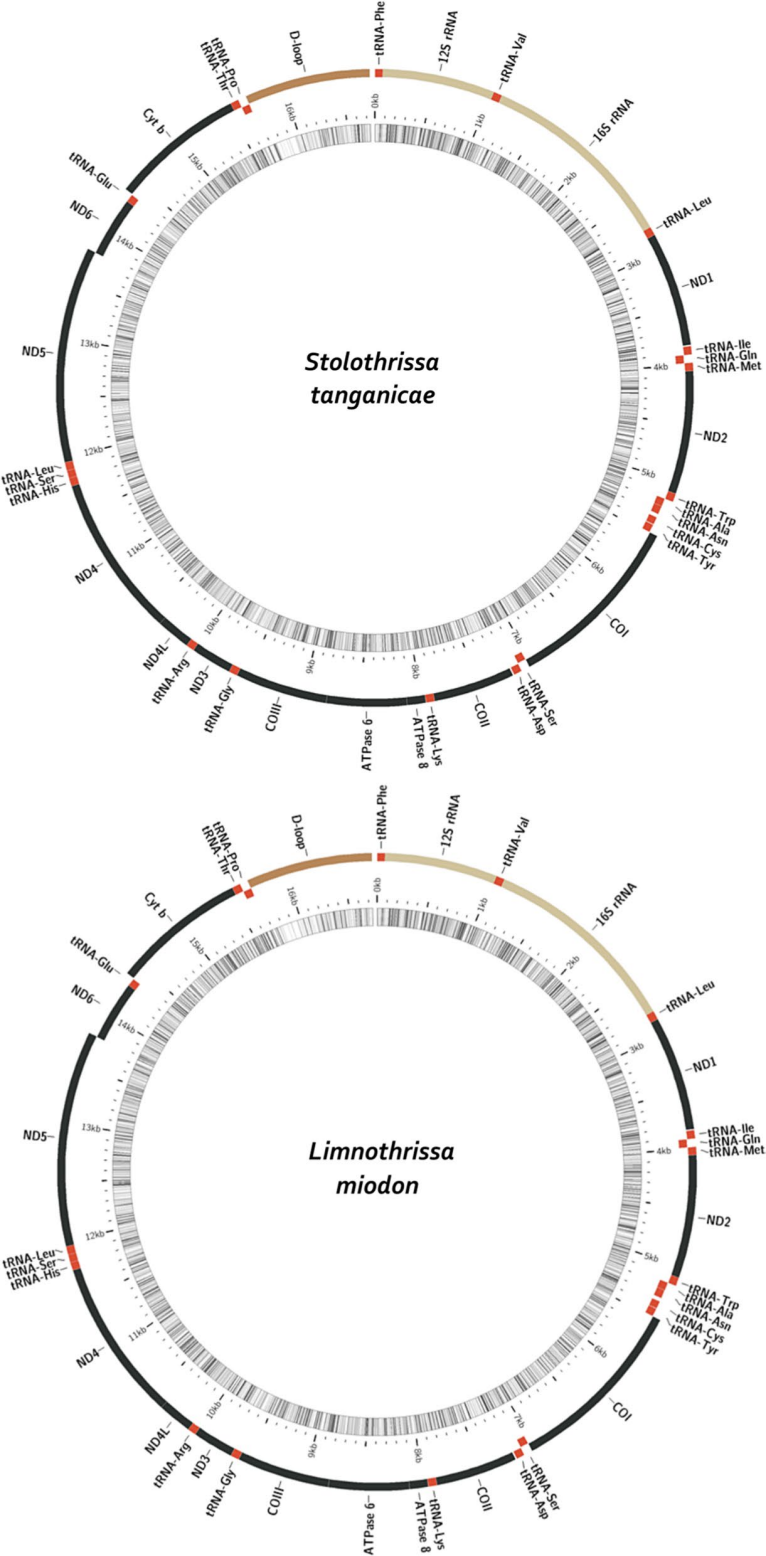
Mitochondrial genomes of *Stolothrissa* *tanganicae* (upper) and *Limnothrissa* *miodon* (lower)*.* Inner circle shading indicates GC-content. Outer circle: black regions are protein coding genes, red regions are tRNA genes, beige regions are rRNA genes, and brown is the D-loop. Regions closer to the centre are located on the minus strand

The mitogenome assemblies of *S.* *tanganicae* and *L.* *miodon* were 16,737 bp and 16,739 bp long, respectively. We annotated all 13 PCGs, 22 transfer RNA (tRNA) genes, 2 rRNA genes (total = 37 genes) as well as the control region (D-loop) in both assemblies in the typical fish and vertebrate mitochondrial gene order [32] (Fig. 1). Gene order analysis in CREx confirmed that all included clupeiforms follow the same gene order, except *Ilisha* *elongata,* where tRNA-Pro and tRNA-Thr appeared transposed.

**Fig. 2:**
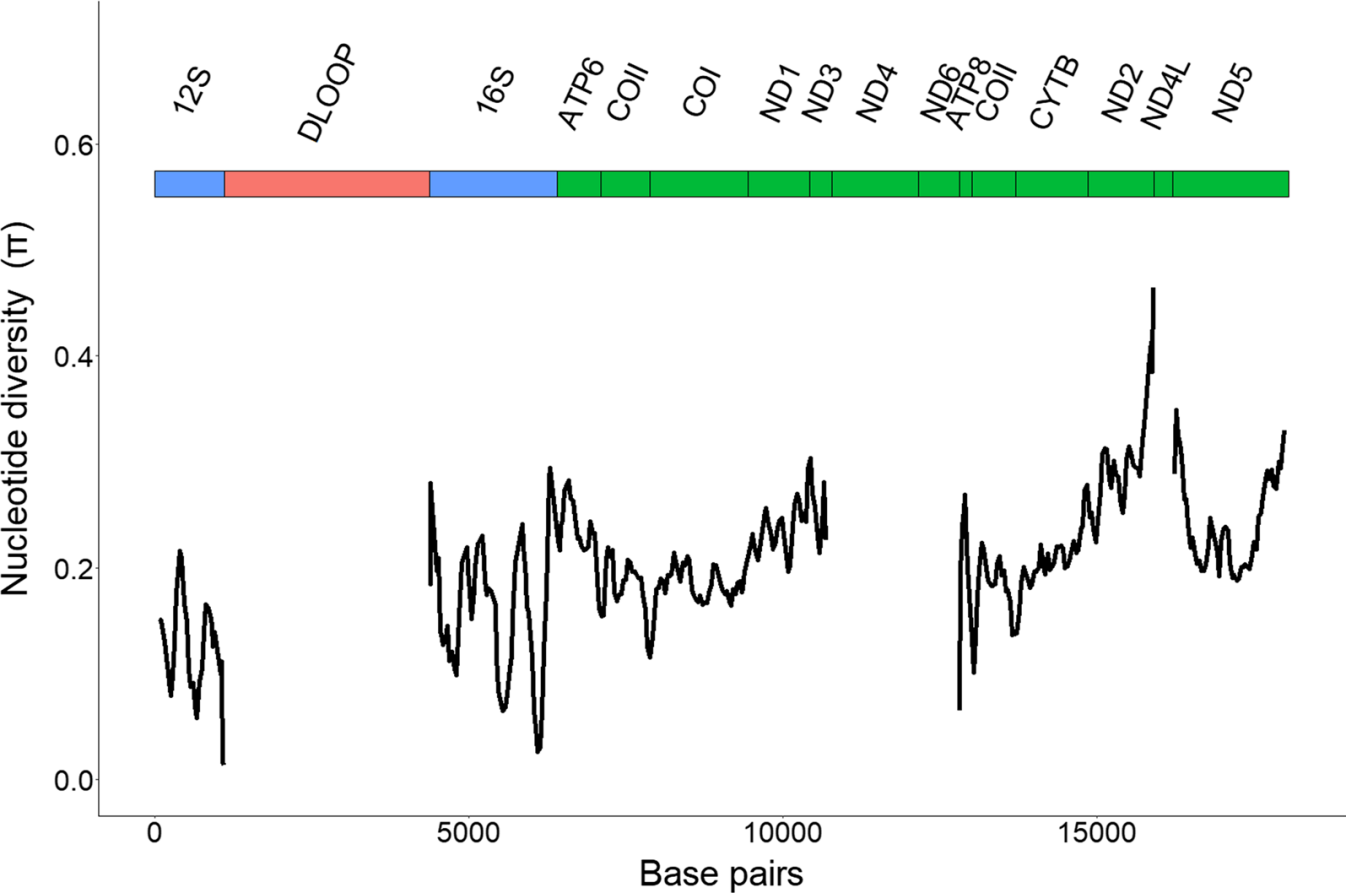
Nucleotide diversity (π) at mitochondrial PCGs (green) and rRNA genes (blue). π was calculated for 107 clupeiform and 6 non-clupeiform fish species using a sliding window with a window size of 150 bp and steps of 35 bp. Values could not be calculated for the gene coding for ND4, ND6, ND4L and for the D-loop (red)

Nucleotide diversity (π), calculated based on alignments of mitochondrial genes between 107 clupeiform and 6 non-clupeiform fish species, showed peaks in the beginning and end of 16S rDNA, as well as in the genes coding for ND1, ND2, ND3, ND5 and ATP synthase membrane subunit 6 (ATP6) (Fig. 2). The genes coding for COI, COII, COIII and CYTB, along with some regions of 12S and 16S rDNA, were relatively less diverse. The alignments for the D-loop, ND4L, ND4 and ND6 genes contained too many gaps to accurately calculate nucleotide diversity and were excluded from this analysis.

Analysis of pairwise genetic distance of PCGs revealed that *S.* *tanganicae* and *L.* *miodon* were among the 0.3% (PCGs) or 3% (non-coding regions) most similar species of Clupeiformes (17th or 206th most similar out of 5778 pairwise comparisons, respectively), and were the two most similar species of Pellonulini (1st out of 28 pairwise comparisons). When considering non-coding regions, the Tanganyika species pair was only the 22nd most similar, with *L.* *miodon* being more similar to most other pellonulines than to *S.* *tanganicae*. Among-group comparisons showed that *L.* *miodon* and *S.* *tanganicae* were almost equally differentiated from the remaining pellonulines when considering PCGs only (distance ± SE for *S.* *tanganicae* = 0.208 ± 0.006, *L.* *miodon* = 0.210 ± 0.006), but that *S.* *tanganicae* was more than twice as differentiated when considering non-coding regions only (distance ± SE for *S.* *tanganicae* = 0.221 ± 0.011, *L.* *miodon* = 0.106 ± 0.005).

### Phylogenetic analysis

**Fig. 3:**
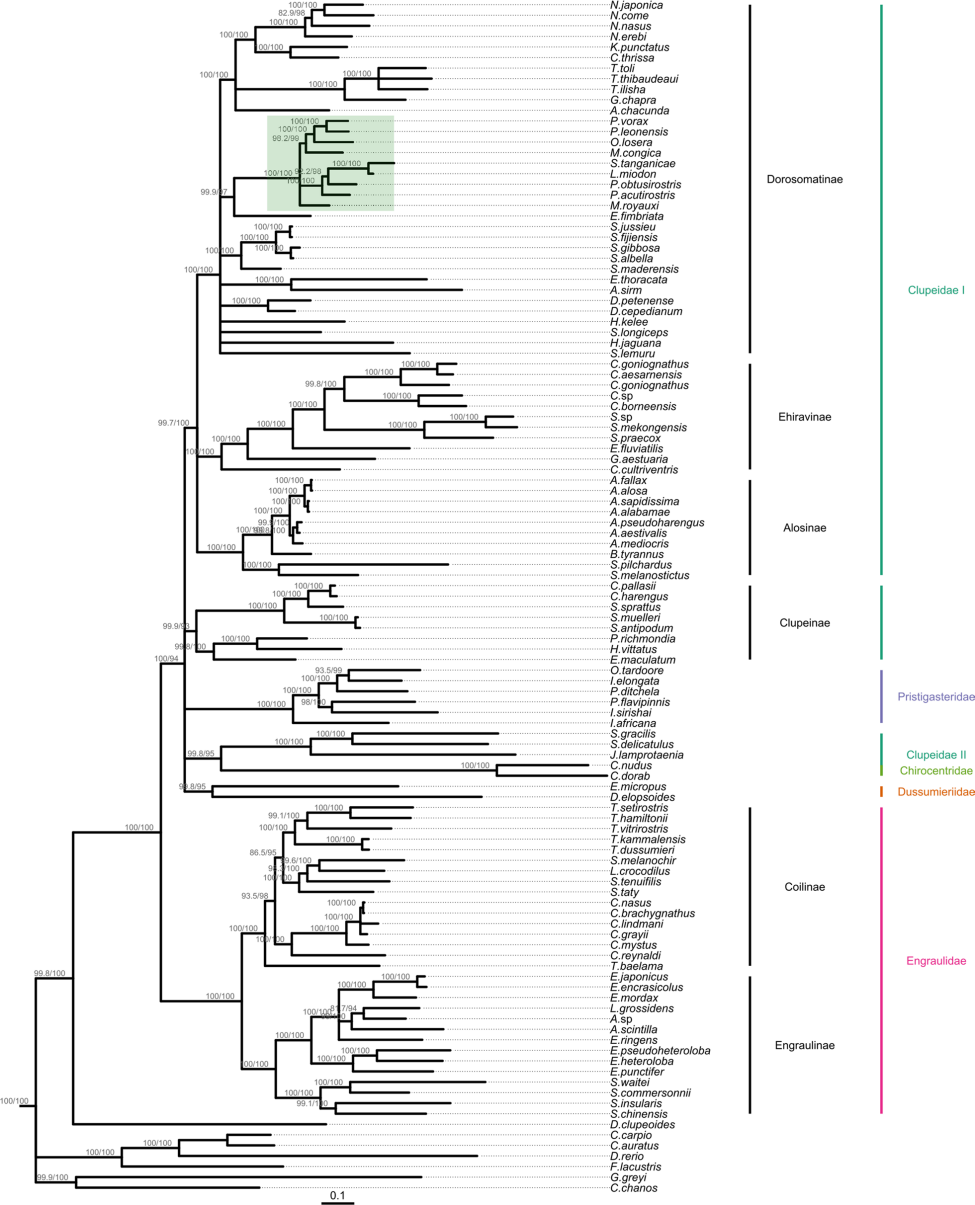
Outgroup-rooted maximum likelihood phylogeny of Clupeiformes. Topology and branch lengths were estimated based on mitochondrial protein-coding genes, rRNA genes and D-loop sequence of 107 clupeiform and 6 non-clupeiform fishes. Node support was assessed by Shimodaira-Hasegawa-like approximate likelihood ratio tests (SH-aLRT%) and ultrafast bootstrap (UFBoot%). Nodes with SH-aLRT% < 75 and UFBoot% < 90 were polytomized and their support values are not shown. The scale bar indicates model-corrected evolutionary distance (expected number of nucleotide substitutions per site). Subfamilies are indicated on the right side in black, families in colour. Pellonulini is highlighted in green

**Fig. 4:**
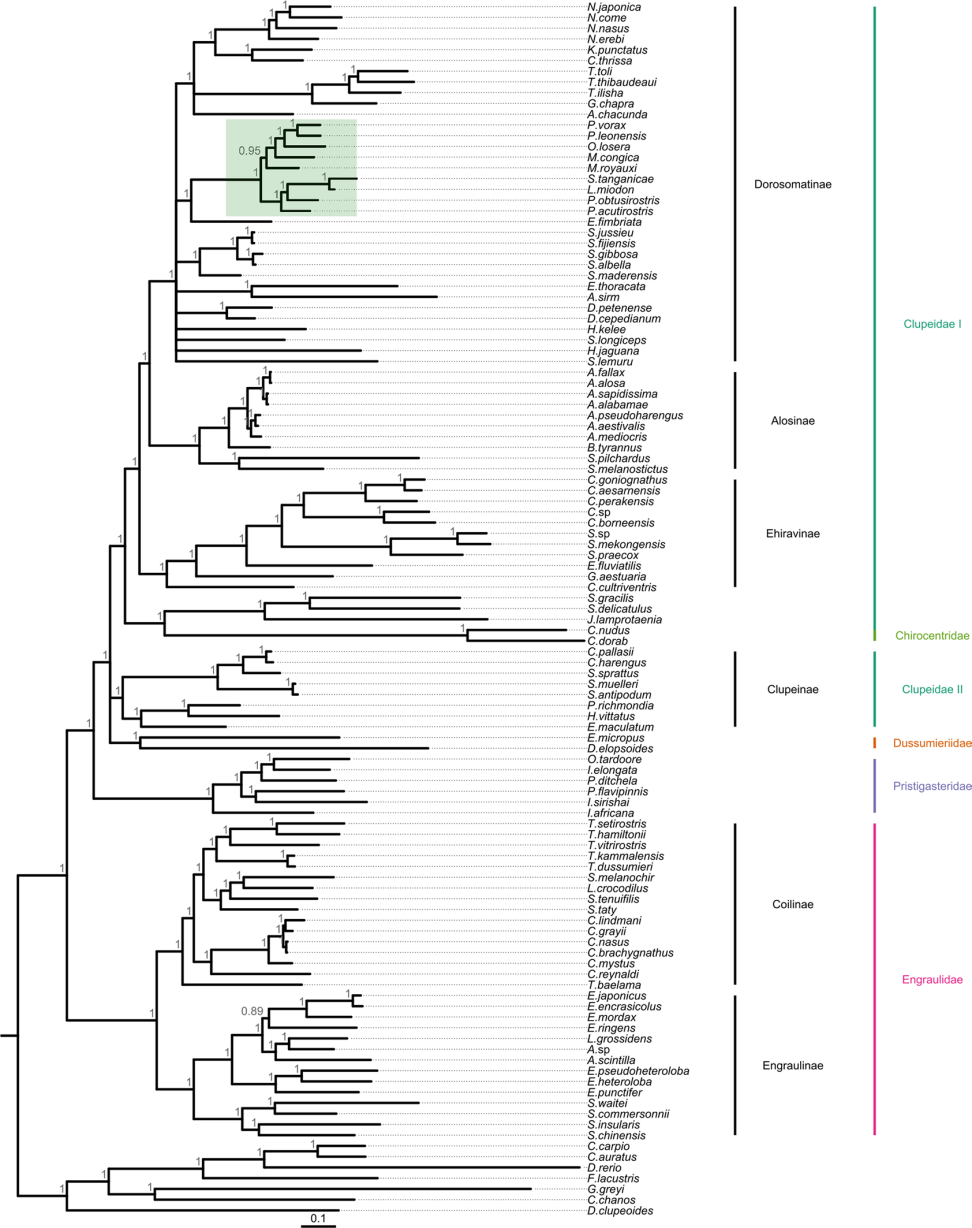
Outgroup-rooted Bayesian phylogeny of Clupeiformes. Topology and branch lengths were estimated based on mitochondrial protein-coding genes, rRNA genes and D-loop sequence of 107 clupeiform and 6 non-clupeiform fishes. Node support was assessed by Bayesian posterior probabilities (BPP). Nodes with BPP < 0.85 were polytomized and their support values are not shown. Probabilities were rounded to the nearest 0.01. The scale bar indicates model-corrected evolutionary distance (expected number of nucleotide substitutions per site). Subfamilies are indicated on the right side in black, families in colour. Pellonulini is highlighted in green

Maximum likelihood (ML) and Bayesian inference (Figs. 3, 4) placed *S.* *tanganicae* and *L.* *miodon* together with the other members of Pellonulini with high statistical support. Within Pellonulini, several genera appeared non-monophyletic. The position of *Microthrissa* *royauxi* was unresolved in these phylogenies, but a Bayesian analysis using only Dorosomatinae placed it paraphyletically with *M.* *congica,* *Pellonula* and *Odaxothrissa* *losera* with high confidence (Additional file 2: Fig. S2). The Lake Tanganyika sardines formed a well-supported clade nested within *Potamothrissa,* with *P.* *obtusirostris* more closely related to the Tanganyika sardines than to *P.* *acutirostris.* In phylogenies resulting from taxon-reduced datasets focusing on Dorosomatinae (Additional file 2), we did not observe any major topological changes compared to those based on the complete dataset. However, some deeper nodes within this subfamily were resolved, such as the placement of *Sardinella* *lemuru* with other species of *Sardinella* (Additional file 2: Figs. S1, S2). In addition, Bayesian inference including all Dorosomatinae and 21 other clupeiforms placed *Gudusia* *chapra* with species of *Tenualosa*, while *Anodontostoma* *chacunda* was placed with *Nematalosa,* *Konosirus* *punctatus* and *Clupanodon* *thrissa.* *Hilsa* *kelee*, *Dorosoma,* *Sardinella* and *Harengula* *jaguana* also clustered together in this Bayesian phylogeny, and *Escualosa* *thoracata* and *Amblygaster* *sirm* formed a well-supported clade (Additional file 2: Fig. S2).

Outside of Dorosomatinae, all subfamilies of Clupeiformes, except Clupeinae, and most of their genera were retrieved with high support. Several deeper node placements in both trees, including some of the traditional clupeiform families with low taxonomic coverage, such as Pristigasteridae, Dussumieridae and Clupeidae II, had low support. Overall, ML and Bayesian analyses were in agreement, with the exception of those deeper, poorly supported nodes. We also found some genera split up or ambiguously placed. For example, there was a closer relationship between *Lycothrissa* *crocodilus* and *Setipinna* *melanochir* than the latter with other species of *Setipinna*. *Thryssa* *baleama* also did not cluster with other representatives of its own genus. *Ilisha* *elongata* clustered with *Pellona* *ditchela* and *Opisthopterus* *tardoore,* while *I.* *africana* and *I.* *sirishai* branched off earlier*.* Only two of the three species of *Sprattus* clustered together. The third, *S.* *sprattus,* was sister to the clade consisting of the two species of *Clupea*.

### Dating of divergence time

**Fig. 5:**
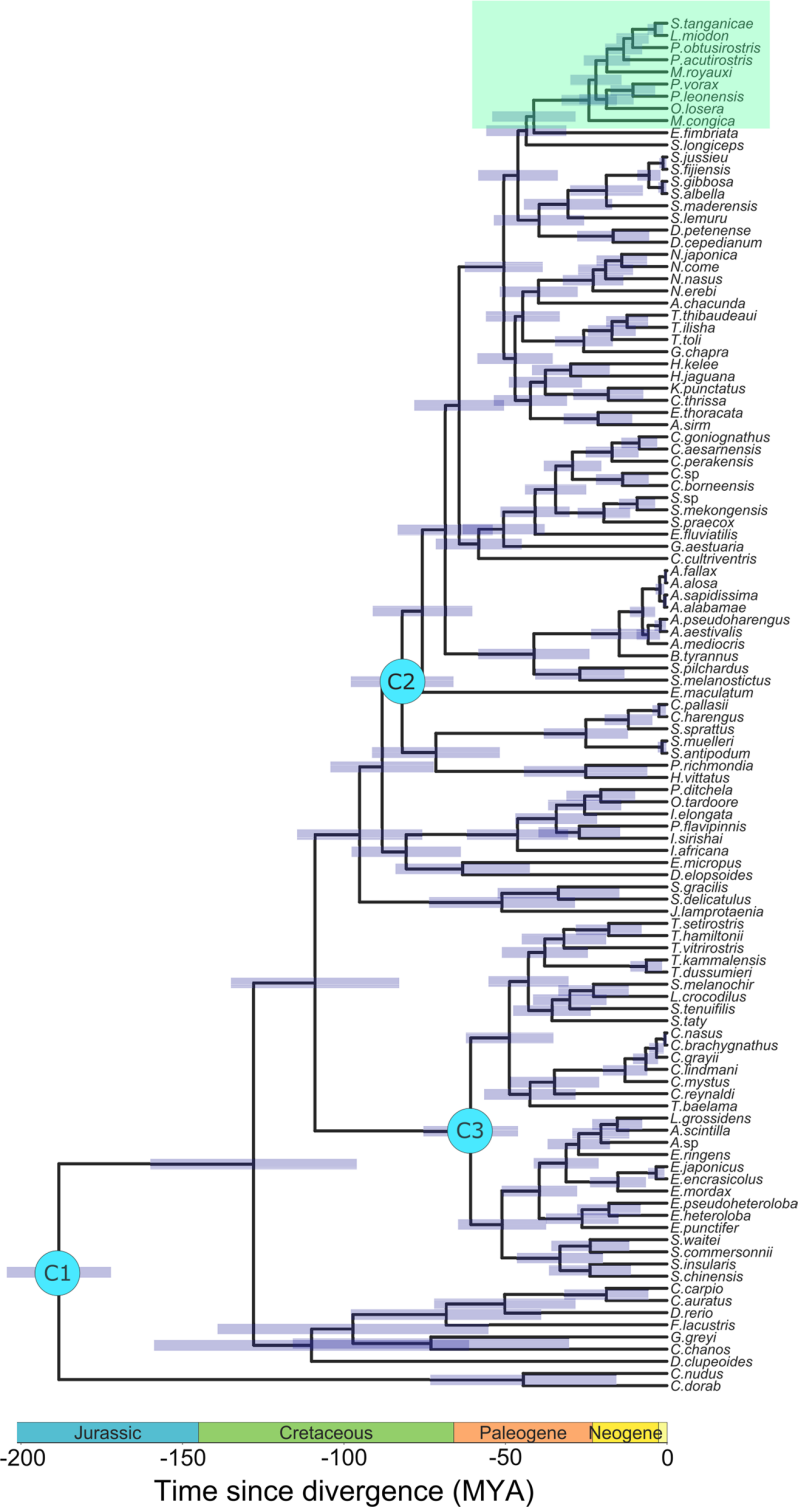
Outgroup-rooted time-calibrated phylogeny of Clupeiformes. Divergence times were estimated using BEAST, based on the first and second codon positions of 13 mitochondrial protein coding genes of 107 clupeiform and 6 non-clupeiform fishes. Blue bars represent Bayesian 95% credibility intervals. Calibration points (C1–C3) are indicated on the corresponding nodes. Pellonulini is highlighted in green

Bayesian analysis estimated the divergence time of the Lake Tanganyika sardines at 3.64 MYA [95% CI: 0.99, 6.29] and the divergence between the most recent common ancestor (MRCA) of the Tanganyika sardines and their closest living relative in the tree, *P.* *obtusirostris,* at 10.92 MYA [95% CI: 6.37, 15.48]. The split between Pellonulini and the other clupeids and thus the timing of a large marine incursion into north-western Africa, was estimated at 43.71 MYA [95% CI: 31.79, 55.63] (Table 1, Fig. 5).

**Table 1:** Comparison of key divergence times, taxa and markers in the pellonuline phylogeny between studies

	Divergence time estimate (MYA)				
	This study	Egan et al. 2018	Bloom and Lovejoy 2014	Wilson et al. 2008	Lavoué et al. 2013
Markers	**PCGs**	**CYT-B**, 16S, rag1, rag2, slc, zic1	**16S, CYT-B,** rag1, rag2	**16S, 12S, CYT-B**	PCGs, tRNAs, rRNAs
Number of taxa (excl. outgroup)	107	190	153	49	82
Number of sites (bp)	18,279	7135	5211	1049–1811	10,733
Node					
*Limnothrissa* *miodon—Stolothrissa* *tanganicae*	3.64 [0.99–6.29]	3.91 [1.19–6.64]	6.61 [2.20–11.01]	7.6 [2.1–15.9]	-
LT sardines– *Potamothrissa* *obtusirostris*	10.92 [6.37–15.48]	10.04 [5.62–14.47]	23.35 [16.37–30.33]	–	–
LT sardines– other pellonulines	–	–	–	27 [25.0–53.3]	–
Incursion 1: pellonulines–other clupeids	43.71 [31.79–55.63]^1^	34.30 [25.56–43.03]^1^	47.58 [35.68–59.47]^1^	37 [25.0–53.3]^2^	46.05 [33.38–58.71]^1^
Incursion 2: *Gilchristella–Sauvagella*	–	25.00 [13.39–36.61]	33.92 [18.94–48.90]	20 [7.5–34.4]	–
Ehiravini–Pellonulini	64.57 [50.17–78.97]	70.13 [59.74–83.44]	98.24 [85.02–111.46]	48 [34.0–66.2]	89.02 [80.97–97.08]

Numbers between square brackets indicate 95% credibility intervals. Divergence times from our study were estimated in BEAST, those from other studies were directly reported or extracted from time-calibrated trees using WebPlotDigitizer. Markers indicated in bold were available for both *Stolothrissa* *tanganicae* and *Limnothrissa* *miodon*. PCGs = all mitochondrial protein coding genes, CYT-B = cytochrome B, 16S = 16S rRNA, 12S = 12S rRNA. ^1^Split Pellonulini – Ethmalosa fimbriata. ^2^Split Pellonulini—other clupeids (*E.* *fimbriata* not included in the study)

## Discussion

We used NGS to sequence and assemble the complete mitochondrial genomes of the Tanganyika sardines, *S.* *tanganicae* and *L.* *miodon,* and built a phylogeny of Clupeiformes using full mitochondrial sequences with a focus on the West and Central-African tribe Pellonulini. Based on these complete mitogenomes, we estimated the divergence time of the Tanganyika sardines to investigate the timing of their speciation in relation to the geology of Lake Tanganyika.

### Conserved gene order in Clupeiformes

Generally, mitochondrial gene arrangements have remained stable for long evolutionary times, but rearrangements do occur in many lineages of both invertebrates and vertebrates. Small rearrangements of neighbouring genes, for example clusters of tRNA-genes, and non-coding regions are especially common [33–35]. Several lineages of Actinopterygii are characterized by such rearrangements, but Clupeiformes is not one of them [32]. Our gene order analysis confirmed the conserved arrangement of mitochondrial genes in this order, aside from one transposition of two tRNA genes (tRNA-Pro and tRNA-Thr) in *Ilisha* *elongata.*

### Inconsistent tree topologies within and outside Pellonulini

Both of our phylogenies (ML and Bayesian) support a single common ancestor for all included pellonuline species and recover *Ethmalosa* *fimbriata* as their sister species with high statistical support, consistent with Lavoué et al. [21]. This is in contrast with the results of Egan et al. [20] and Bloom and Lovejoy [19], neither of whom found good support for this sister-species relationship. *Microthrissa* appeared non-monophyletic in our study, and the Tanganyika sardines rendered *Potamothrissa* paraphyletic. The position of *Microthrissa* differs in almost every study that has addressed it. According to Egan et al. [20], *M.* *royauxi* is more closely related to the Tanganyika sardines and *Potamothrissa*, while *M.* *congica* clustered with *Pellonula* and *Odaxothrissa.* In the study of Wilson et al. [17], two specimens of *M.* *royauxi* did not even cluster together, but this may be a taxonomic artefact. Regardless of this possibility, *M.* *congica* was found more closely related to *Pellonula* than to *M.* *royauxi.* Bloom and Lovejoy [19], on the other hand, found both *Microthrissa* species more closely related to members of *Pellonula* and *Odaxothrissa* than to the clade including the Tanganyika sardines and *Potamothrissa.* In accordance, our analysis could not consistently recover the exact position of *M.* *royauxi* with high statistical support, but it did confirm that *M.* *congica* is more closely related to members of *Pellonula* and *Odaxothrissa* than to *M.* *royauxi.* The Lake Tanganyika sardines formed a well-supported clade nested within *Potamothrissa.* Contrarily, our study is the first to place *P.* *obtusirostris* and *P.* *acutirostris* in the same clade, albeit paraphyletically. With the improved resolution resulting from our whole mitogenome approach and the inclusion of a large number of clupeiform taxa, our study also confirms *E.* *fimbriata* as sister species of Pellonulini.

The source of these inconsistent topologies is unclear, but could be related to smaller, more variable and partly incomplete gene datasets in previous studies. Egan et al. [20] included only the gene coding for CYT-B for most members of Pellonulini, including the Tanganyika sardines, and three nuclear genes and/or the 16S rRNA gene for others. Bloom and Lovejoy [19] did not include *O.* *losera* and *P.* *acutirostris* and used CYT-B and 16S rRNA genes for most, and two nuclear genes for two species, while Wilson et al. [17] used only mitochondrial genes (CYT-B, 16S rRNA, 12S rRNA). None of the previous studies included more than around 5 kbp of alignment data, except Lavoué et al. [21], who included all PCG, rRNA and tRNA sequences which amounted to slightly over 10kbp. Taxonomic coverage was also highly variable, ranging from 49 to 190 clupeoid species, the lowest number belonging to Wilson et al. [17], which also had the largest credibility interval but had the highest taxonomic coverage of Pellonulini. The Tanganyika sardines and other pellonulines were also missing from several of these studies. Although the inclusion of taxa with incomplete datasets can help to resolve phylogenies [36], there is a trade-off with increased risk of phylogenetic artefacts, and difficulty detecting multiple substitutions [37, 38]. In contrast, our study had the first nearly complete dataset for all taxa, thanks to the readily available mitochondrial genomes from many pellonuline and other clupeid species.

Morphological diversity is relatively low in representatives of Pellonulini compared to for example cichlids [15, 39]. In the FAO species catalogue of clupeoid fishes, several ambiguous identifications and uncertain species descriptions are mentioned. For instance, the distinction between *O.* *losera* and *O.* *vittata* is based solely on the number of gill rakers, which also varies with the age of the specimen, a common occurrence among clupeid fishes. In *P.* *leonensis,* there is also evidence for undescribed subspecies exhibiting characteristics of both *P.* *leonensis* and *P.* *vorax,* or even specimens belonging to *Cynothrissa* [39]*.* It is thus not inconceivable that misidentifications have confounded past taxonomic studies, and that a taxonomic revision of these genera may be needed.

Outside of Pellonulini, we recovered most of the traditional families and subfamilies of Clupeiformes with high statistical support. The positions of the families with lower taxonomic coverage, including Pristigasteridae, Chirocentridae, Dussumieridae, remained unresolved, Clupeidae was not monophyletic and several species were also placed away from their congeners, for example in the genera *Setipinna*, *Thryssa,* *Ilisha* and *Sprattus.* These latter two findings are consistent with previous studies of Clupeiformes with taxonomic coverage comparable to ours [19, 20, 40], underlining the need for revision of these taxa.

### Inconsistent divergence time estimates in Clupeiformes

Bayesian analysis estimated the divergence time of the Lake Tanganyika sardines at around 3.64 MYA [95% CI: 0.99, 6.29]. This estimate is younger than the previous estimate by Wilson et al. [17] at 7.6 MYA, but within its credibility interval [95% CI: 2.1, 15.9]. Conversely, their estimate fell just outside our credibility interval. Our estimate is also younger compared to the one by Bloom and Lovejoy [19] at 6.61 MYA [95% CI: 2.20, 11.01], but in accordance with Egan et al. [20] at 3.91 MYA [95% CI: 1.19, 6.64]. Our credibility intervals were smaller than those of Wilson et al. [17], but comparable to Bloom and Lovejoy [19] and Egan et al. [20]. Other, deeper nodes of interest also differed between the studies. Overall, our estimates most closely agreed with those of Egan et al. [20], except for the divergence time of the pellonulines from other clupeids, which was around 10 MYA older in our study. Bloom and Lovejoy [19] found consistently older estimates, while Wilson et al. [17] estimated the more recent nodes as older, and the deeper nodes as younger than the other three studies.

The differences in estimated divergence times between the studies can be partially attributed to the different estimation procedures. Specifically, methodological choices for Bayesian dating of nodes can strongly influence the accuracy and precision of the divergence time estimates, for example the choice of priors to account for uncertainty surrounding the age of a fossil, and the choice of clock model [31, 41]. Almost all the studies we compared here dated divergence using a fossil-calibrated uncorrelated (relaxed) clock model implemented in BEAST, accounting for substitution rate heterogeneity among branches. Six to eight fossil calibrations were specified as exponential priors with soft maximum ages. Only Wilson et al. [17] used an autocorrelated clock approach with seven fossil calibrations specified as uniform priors. In accordance with the three more recent studies on the divergence times of Clupeiformes [19–21], but in contrast with Wilson et al. [17], we chose a relaxed clock model, in accordance with the varying speeds of diversification in different clupeid lineages [40]. A possible caveat of our divergence time dating analysis is the exclusion of highly variable sequences of the D-loop region, rRNAs and third codon positions, which was necessary to achieve convergence of the model. These sites may be useful to resolve ambiguous recent divergences [42] and produce different, most likely slightly older, divergence time estimates.

Ideally, time-calibration of the diversification of the pellonulines would be based on fossils within this clade. Unfortunately, there are no known pellonuline fossils, and the fossil record of fishes of Central Africa in general is sparse [16]. In contrast to Wilson et al. [17], Bloom and Lovejoy [19] and Egan et al. [20], we decided to use pre-calibrated time scaling points (“secondary calibration”) from a previously published study [43] instead of direct fossil calibration (“primary calibration”). Secondary calibrations can result in younger or older, depending on their placement, and falsely narrowed estimates of node ages [38, 44], but were necessary in our case to achieve convergence. Some caution is warranted when interpreting the width of our confidence intervals, but for the reasons described in the methods section ‘Dating of divergence time’, we are confident that our methodological choices have produced node age estimates with the best possible accuracy. The robustness of our approach is further supported by correspondence of our estimates to fossil ages from the literature. The ancestor of Clupeoidei was estimated at 127.48 [95% CI: 95.70, 159.27] in our study, older than the minimum age of 125 MYA attributed to the fossil of **†***Cynoclupea* *nelsoni* [45]. The MRCA of Engraulidae was estimated at 60.10 MYA [95% CI: 44.70, 75.50], corresponding to the minimum age of the fossil of **†***Eoengraulis* *fasoloi* of 50 MYA [46]. *Dorosoma* *petenense* was estimated as relatively old at 17.22 MYA [95% CI: 5.46, 28.97], but again within the boundaries of the minimum age of 2.5 MYA of the oldest fossil of *Dorosoma* *petenense* [47].

### Utility of whole mitochondrial genomes for phylogenomic analysis and divergence time dating

Mitochondrial protein coding genes vary in their ability to recover known phylogenetic topologies. The sequences of ND4, ND5, COI and CYTB genes are generally useful for phylogenetic questions, while fast evolving genes such as ND4L and ATP8 are regarded as poor phylogenetic performers, although this differs per study and taxon [25, 26]. Indeed, we found relatively high nucleotide diversity in some genes or regions compared to others, including parts of the genes coding for the ATP6, ND1, ND2, ND3 and parts of ND5. However, whole mitochondrial genomes can recover accurate phylogenies with high resolution, despite containing “poor” phylogenetic performers [27, 48]. A smaller subset of “good” mitochondrial genes may be able to recover the same topology as the entire mitochondrial genome, but this is highly taxon-specific [25–27, 48]. Thus, utilizing more markers that provide complementary information is preferable if previous taxon-specific information on the utility of single markers is not available [27].

Phylogenies based on mitochondrial DNA (mtDNA) alone come with limitations [24, 49]. First, mtDNA is subject to frequent introgression, horizontal gene transfer, incomplete lineage sorting, and mitochondrial capture. As a result, past hybridization can go undetected in the absence of nuclear or morphological data [24, 50–54]. Second, sequencing or assembling nuclear pseudogenes of mitochondrial origin into mitogenomes can introduce false polyphyly within species or closely related taxa [55]. Third, the fast substitution rates of mtDNA make accurate estimation of deep divergences difficult due to problems with saturation and ensuing homoplasy [26, 27]. Overall, nuclear and mtDNA data can contrast or complement each other both in terms of tree topology and branch lengths [7, 8, 40]. Inconsistencies between them should be considered informative, and future studies should strive to include both data sources to reconstruct more complete evolutionary scenarios [49].

There are several examples of ongoing hybridization between clupeid species [56–59]. With their similar habitat, nursery areas, and modes of reproduction, the Tanganyika sardines may well have a history of introgression that has remained concealed here. At the start of the Eocene (around 50 MYA), global sea levels were more than 100 m higher than today, and steadily decreased over the next 20 million years, with smaller maxima in between [16, 60, 61]. These fluctuations may have allowed frequent isolations and reconnections in the Congo Basin, favouring hybridization between other newly formed pellonuline species as well. To completely resolve the species tree of Pellonulini, phylogenomic analyses using nuclear genomic markers and multiple individuals per species are needed (but see Bloom & Egan [40], who found similar divergence time estimates with mtDNA and nuclear DNA datasets).

### Updated divergence time suggests intralacustrine speciation of the Tanganyika sardines

Present-day distributions of several Afrotropical freshwater fish lineages show striking overlap, including members of Pellonulini, Kneriidae and Phractolaemidae, providing evidence for a single marine-freshwater transition across West- and Central Africa around 50 MYA during a period of high sea levels [16]. Despite the high sea levels, Lake Tanganyika was likely never in direct contact with the ocean and has not experienced much higher water levels than at present [3, 6]. Furthermore, due to uplift of the borders of the Congo Basin from the Cenozoic onwards, the possibility of an additional marine incursion close to the lake is faint [18]. It is therefore more likely the Lake Tanganyika sardines evolved from riverine clupeids. Indeed, the presence of a large body of water covering a large area of the Congo Basin (“paleo-lake Congo”) until the Pliocene or early Pleistocene (2–12 MYA, [62, 63]), may have increased the connectivity between the Congo tributaries and its surrounding lakes, and may have facilitated the entry of riverine species into the predecessor of Lake Tanganyika at this time.

Our improved divergence time estimates of the Tanganyika sardines (3.64 MYA) and their MRCA from other pellonulines (10.92 MYA) help us to better understand their origin and colonisation time in connection to the geological history of the lake. Our estimates are compatible with (1) the entrance of the MRCA of the Tanganyika sardines into the newly formed Tanganyika basin (around 12 MYA) via the tributaries of the proto-Malagarasi-Congo River; and (2) intralacustrine speciation at the onset of deep- and clearwater conditions after the sub-basins fused (5–6 MYA). However, based on the 95% credibility intervals of our estimates, we cannot exclude the possibility that the MRCA of the Tanganyika sardines diverged from *P.* *obtusirostris* outside of the proto-lake and entered it sometime between the time of its formation and the fusion of its sub-basins.

Which environmental conditions triggered the divergence between *S.* *tanganicae* and *L.* *miodon* remains uncertain. Sexual selection, such as in cichlids [64], is unlikely to have played a large role due to the mode of reproduction of the clupeids. Ecological differences can be powerful drivers of speciation, even in (partial) sympatry [65, 66]. The newly fused basin, adding ecological heterogeneity to the ancestral sardine’s environment, may have favoured dietary specialization through divergent selection on polymorphic trophic traits. Niche separation and divergence can then prompt genetic reproductive isolation if reinforced by spatial or temporal separation of spawning or lower hybrid fitness [65–67]. Indeed, contemporary populations of *L.* *miodon* seem to spawn all year round and mostly in the littoral, while populations of *S.* *tanganicae* exhibit clear peak spawning times in the pelagic [68]. This suggests that at some point during their divergence, spawning became more common in their respective preferred habitats. An alternative explanation is that their speciation was triggered by periods of allopatry [65, 67]. Given our credibility intervals and the frequent water-level fluctuations potentially separating and reconnecting the southern and central sub-basins of Lake Tanganyika several times, it is likely that ancestral sardine populations frequently occurred in partial or complete isolation.

### A *Limnothrissa-*like ancestor of the Tanganyika sardines?

According to our ML and Bayesian phylogenies, the Tanganyika sardines are most closely related to *P.* *obtusirostris,* *P.* *acutirostris*, and *M.* *royauxi*, but not *M.* *congica*, the closest living relative being *P.* *obtusirostris*. Ecological studies of these species are sparse, hindering systematic comparison. Much of their distribution overlaps and, aside from *M.* *royauxi*, stretches across most of the Congo Basin all the way down to the Lukuga River, which was connected to the Malagarasi River east of Tanganyika around the time of the lake’s formation. While *M.* *congica* and *P.* *acutirostris* occur in both rivers and lakes, *P.* *obtusirostris* and *M.* *royauxi* seem to be more strictly riverine [39]. The diet of *P.* *obtusirostris* and *M.* *congica* consists mostly of aquatic and terrestrial insects [39, 69], with occasional piscivory in *M.* *congica* [69]. In *M.* *congica*, strong seasonal effects of water level fluctuations on both diet and reproduction have been observed [69, 70]. Ecologically, *L.* *miodon*, with its generalist diet including insects and small fishes, is more similar to the riverine pellonulines than *S.* *tanganicae*, which is a strict planktivore. In addition, individuals of *L.* *miodon* and species of *Potamothrissa* share a morphological feature that is otherwise rare in clupeid fishes: a row of saw-like teeth at the side of the lower jaw [39]. We thus suggest that the ancestral Tanganyika sardine shared more ecological traits with *L.* *miodon* than with *S.* *tanganicae.* This is also reflected in the more shorebound and generalist lifestyle of *L.* *miodon*, and its ability to invade the Cahora Bassa reservoir though dispersal via the riverine environment of the Zambezi [71], suggesting a relatively high ecological flexibility compared to *S.* *tanganicae* [72], and thus a higher ability to colonize a new environment. Nevertheless, the presence of established contemporary populations of *S.* *tanganicae* in one of the Congo’s tributaries, the Lukuga, attest its ability to inhabit, or at least cross, non-pelagic environments, provided the water composition is sufficiently similar [73]. We also found larger genetic differentiation of *S.* *tanganicae* than *L.* *miodon* from the remaining pellonulines in non-coding regions. This could further support our hypothesis of a higher relatedness between *L.* *miodon* and the ancestral sardine, but may also indicate different demographic histories [74]. Kmentová et al. [75] found signatures of recent population expansion in both *L.* *miodon* and *S.* *tanganicae*, but these were more pronounced in the latter. The population expansion in *S.* *tanganicae* might be linked to the fusion of sub-basins, or any other major lake-level fluctuation that increased the amount of pelagic habitat. Similarly, species of the pelagic cichlid tribe Bathybatini showed recent demographic expansions, probably also linked to lake-level fluctuations [76].

## Conclusion

Using NGS data, we assembled and annotated the full mitochondrial genomes of the Tanganyika sardines *S.* *tanganicae* and *L.* *miodon.* Putting them into phylogenetic context with full mitochondrial genomes of 107 other clupeiform species, we estimate their divergence time at 3.64 MYA, and divergence from their riverine ancestor at 10.92 MYA. This estimate implies that the MRCA of the Tanganyika sardines entered Lake Tanganyika shortly after its formation during a period of high connectivity of the Congo Basin’s water bodies. We suggest that the speciation event is likely to have been brought on by the fusion of Lake Tanganyika’s sub-basins and the subsequent clearwater conditions.

The mitochondrial genomes of *S.* *tanganicae* and *L.* *miodon* are valuable resources for future studies of the evolutionary history of these species at the population level, for example as a reference for barcoding, studies of their mitochondrial diversity and evolutionary history, as well as macroevolutionary study of relationships within Pellonulini and Clupeiformes. Future work should focus on the divergence time of different regions of the Tanganyika sardines’ genomes and compare them to a dataset of nuclear genes or genome-wide data. This, in combination with formal tests for hybridization, could help to gauge the role of introgression in the timing and the scenario of speciation. Nuclear genomic sequences from several individuals of all members of Pellonulini would allow a more precise reconstruction of their colonization of West-Africa and clarify the ambiguous classifications in this group.

## Methods

### DNA extraction, library preparation and sequencing

One female individual of the two species was collected from liftnet fishing catches on the night of 15th of December 2018 off the shore of Uvira, Democratic Republic of Congo. Fish were dissected to extract liver tissue, which was directly frozen on dry ice and subsequently stored at − 20 °C until extraction. High molecular weight genomic DNA (gDNA) was extracted using a Blood and cell culture DNA Midi Kit (Qiagen). Libraries were prepared for each species separately using Chromium Genome Library & Gel Bead Kit v.2 (10X Genomics, cat. 120258), Chromium Genome Chip Kit v.2 (10X Genomics, cat. 120257), Chromium i7 Multiplex Kit (10X Genomics, cat. 120262) and Chromium controller according to the manufacturer’s instructions with one modification (added shearing step before Illumina library preparation). Briefly, gDNA diluted to 1.02 ng/μl was combined with Master Mix, a library of Genome Gel Beads, and partitioning oil to create Gel Bead-in-Emulsions (GEMs) on a Chromium Genome Chip. The GEMs were isothermally amplified with primers containing an Illumina Read 1 sequencing primer, a unique 16 bp 10X barcode and a 6 bp random primer sequence. Barcoded DNA fragments were recovered for Illumina library construction. The amount and fragment size of post-GEM DNA was quantified using a Bioanalyzer 2100 with an Agilent High sensitivity DNA kit (Agilent, cat. 5067-4626). Prior to Illumina library construction, the GEM amplification product was sheared on an E220 Focused Ultrasonicator (Covaris, Woburn, MA) to approximately 350 bp (55 s at peak power = 175, duty factor = 10, and cycle/burst = 200). Then, the sheared GEMs were converted to a sequencing library following the 10X standard operating procedure. The library was quantified by qPCR with a Kapa Library Quant kit (Kapa Biosystems-Roche) and sequenced on a partial lane of the NovaSeq6000 sequencer (Illumina, San Diego, CA) with paired-end 150 bp reads.

### Mitochondrial genome assembly

For mitogenome assembly, raw 10X Chromium reads were processed using the proc10xG package [77]. Process_10xReads.py was run using default settings to remove GEM and individual sample barcodes. The resulting reads passed read assessment by FastQC v0.11.7 [78] without any quality problems, residual adapters or overrepresented sequences, the latter also commonly indicating adapter contamination. Mitogenomes of the two sardines were assembled from these barcode trimmed reads using MitoZ v.2.4-alpha [79] with default settings. Mitochondrial genes were annotated using the Mitofish annotator web service [80].

### Taxonomic sampling and alignment

Taxonomic sampling for phylogenetic analysis included all members of Clupeiformes for which a complete mitochondrial genome is published (accessed 21st of October 2020, Additional file 1: Table S1). The sequences of *Odaxothrissa* *vittata* (NC_009590.1) and *Etrumeus* *teres* (NC_009583.1) were identical to *Pellonula* *vorax* and *E.* *micropus,* respectively, and were omitted from analysis. The outgroup was selected based on Lavoué et al. [21] and includes the denticle herring (*Denticeps* *clupeoides*), two alepocephaliforms, four ostariophysians and two euteleosts (Additional file 1: Table S1). We extracted mitogenomes and their annotations from NCBI using a combination of efetch [81] and custom Bash, Perl and Python scripts. We manually verified the new annotations of *S.* *tanganicae* and *L.* *miodon* by comparing the nucleotide and amino acid sequences, translated using vertebrate mitochondrial code, to the already published pellonuline mitogenomes, and checking for the presence of start and stop-codons at the appropriate positions in MEGA-11 [82].

We separately aligned each PCG using a codon-based MAFFT algorithm in the TranslatorX server [83]. We selected options for less stringent selection which allowed smaller final blocks with gap positions and less strict flanking positions. D-loop sequences were aligned using MAFFT v.7.470 with default parameters, and sequences of the rRNA coding regions using MAFFT with the -qinsi option [84]. Incomplete or missing regions were coded as missing data (N). We performed all alignments with and without alignment cleaning by Gblocks, further referred to as ‘complete’ and ‘trimmed’ alignments. We used AMAS v.0.98 [85] to separately concatenate the trimmed and complete alignments, producing one trimmed and one complete dataset. The phylogenetic content of these datasets was compared using likelihood mapping [86] implemented in TREE-PUZZLE v.5.3.rc16 [87]. We determined the optimal model for each dataset using jModelTest 2 [88] and specified these as input models for TREE-PUZZLE. Since there was no difference in phylogenetic content (86.6% fully resolved quartets, 2.3% partly resolved, 11.1% unresolved), we performed all subsequent analyses on the complete dataset of 18,279 bp.

### Genetic diversity, divergence, and gene order

We calculated nucleotide diversity (π) of the final alignment using a sliding window analysis implemented in DnaSP v.6 [89] with a window size of 300 bp and steps of 15 bp. We used MEGA to quantify divergence between species and clades. We calculated pairwise genetic distances between all species, and mean between-group genetic distances between *S.* *tanganicae,* *L.* *miodon* and the remaining members of Pellonulini and Clupeiformes (in each of these comparisons excluding the other Tanganyika clupeid). The distances were calculated separately for PCGs (vertebrate mitochondrial code) and non-coding regions using a Tamura-Nei model including transitions and transversions, gamma-distributed rate variation among sites and heterogenous rate patterns among lineages. Gaps and missing data were deleted in a pairwise manner. The gamma parameter was estimated separately for the PCG and non-coding dataset using jModelTest 2. To estimate the relative similarity of *S.* *tanganicae* and *L.* *miodon* compared to similarities among other clupeids, we ranked all pairwise genetic distances of (1) Clupeiformes and (2) Pellonulini and calculated in which percentile the Tanganyika sardines fell using R v.4.0.4 [90]. Finally, we compared the gene order of all species included in our study using the CREx web application [91]. Two species, *Alosa* *fallax* and *Hilsa* *kelee,* were missing several markers (genes), and were thus excluded from this analysis.

### Phylogenomic tree building

We ran IQ-TREE v.1.6.12 [92] twice on the complete dataset. The first run determined the best partition scheme (option -m MF + MERGE), allowing different models of molecular evolution in different genes/regions and, for PCGs, at the different codon positions [93]. The second run first implemented ModelFinder [94] to find the optimal model of evolution for each partition found by the previous round (options -spp and -m MFP), then constructed a ML tree, and finally assessed nodal support by 10,000 ultrafast bootstraps (generating support value UFBoot%) and 1,000 Shimodaira-Hasegawa-like approximate likelihood ratio test replicates (generating support value SH-aLRT%). A clade can be considered well supported if UFBoot% ≥ 95 (corresponding to a ~ 95% chance that the clade is true) and SH-aLRT% ≥ 80 [95, 96].

Using the IQ-TREE-derived partition and models, we constructed a Bayesian phylogeny in MrBayes v.3.2.7a [97], allowing estimation of the model parameters for each partition separately (unlinked character state frequencies, substitution rates of the GTR + I + G model). Two independent runs with 4 MCMC chains ran for 60 million generations, sampling every 500 generations and discarding the first 25% as burn in. The remaining samples were used to calculate Bayesian posterior probabilities (BPP) for each node in order to assess nodal support. A clade is considered well supported if BBP ≥ 0.9. The models converged, as indicated by the average standard deviation of split frequencies approaching zero, the absence of a trend in log likelihood of the runs, an Effective Sample Size (ESS) > 200, and the Potential Scale Reduction Factor approaching 1 [98].

### Dating of divergence time

We estimated branch lengths and divergence times between *S.* *tanganicae* and *L.* *miodon* and five other nodes of interest (Table 1) using Bayesian relaxed molecular clock analysis implemented in BEAST v.2.6.3 [99] with the tree topology from MrBayes as a starting tree. We conducted four independent BEAST runs of 50 million generations. Convergence was ensured by checking if ESS was higher than 200 for all parameters using Tracer v.1.7.1 [100]. Trees were summarized and annotated using the TreeAnnotator module in BEAST. All final trees were visualized using ggtree v.3.3.0.901 in R [101].

For the time calibration of our tree, we first attempted a “primary calibration”, which relies solely on fossils, using the complete dataset. However, when this model failed to converge, even after hundreds of millions of generations, we modified our analysis in two ways. First, instead of primary calibration, we chose to apply “secondary calibration”, which uses estimates from an already existing phylogeny. We used three calibration points from a recent phylogeny of the teleosts using more than 30 fossils [43]. We included the MRCA of members of Clupeiformes and our outgroup (including *Danio* *rerio,* *Cyprinus* *carpio* and *Chanos* *chanos*) at 194 MYA (**C1**), MRCA of members of Clupeinae and Alosinae at 73 MYA (**C2**), and MRCA of Engraulidae at 61 MYA (**C3**). The calibration points were implemented as normal prior distributions for the node ages in BEAST. Secondary calibration inevitably incorporates geological and fossil uncertainty along with uncertainties associated with the primary dataset. They tend to push node age estimates into the more recent direction and falsely narrow the credibility intervals, especially when using a single old secondary calibration [38, 44], but see Powell et al. [102]. In our case, we presume the associated problems to be minimal for four reasons. (1) We used secondary calibrations for both old and younger nodes, which should diminish the tendency to estimate other nodes as younger [44]. (2) In Bayesian analysis implemented in BEAST, lognormally or exponentially distributed prior distributions are most commonly used for primary calibrations. These produce younger node age estimates and narrower credibility intervals than uniform (and presumably normally distributed) priors [44], which are more commonly used for secondary calibrations. (3) We validated the robustness of our secondary calibration by comparing the results of the BEAST dating to primary fossil calibration points from the literature, see discussion section ‘Inconsistent divergence time estimates in Clupeiformes’. (4) The true uncertainty associated with secondary calibrations from Hughes et al. [43] based on more than 30 well-characterized fossils is likely smaller than that produced by using only three or four primary fossils with lognormal distributions with large variance.

Second, we omitted the hypervariable D-loop sequence, regions coding for rRNA, and the third codon positions, leaving a site-reduced dataset with only first and second codon positions of all PCGs. We also merged the separate partitions found by IQ-TREE into two partitions containing first and second codon positions. Since variable regions can be informative for estimating recent divergences, we repeated the analysis with two taxon-reduced datasets focusing on Dorosomatinae, in which we kept these variable regions (Additional file 2).

Finally, we compared our divergence time estimates for six nodes of interest with published estimates [17, 19–21] (Table 1). Estimates and 95% CI that were not directly reported in these publications were extracted from time-calibrated trees using WebPlotDigitizer v.4.5 [103].

## Supplementary Information

**Additional file 1.** Taxonomic information, accession numbers and references of mitochondrial genomes used for phylogenetic analyses.

**Additional file 2.** Phylogenetic analysis and divergence time dating of taxon-reduced datasets focusing on Dorosomatinae.

## Data Availability

The datasets supporting the conclusions of this article are available in the NCBI Sequence Read Archive [http://www.ncbi.nlm.nih.gov/bioproject/860551] and GenBank [Accessions: OP022425, OP021863] repositories. Custom scripts are available on Github: https://github.com/lmilec/mitoprep.

## Abbreviations

MYA: Million years ago
COI: Cytochrome *c* oxidase subunit I
CYTB: Cytochrome B
rRNA: Ribosomal RNA
ND: NADH-ubiquinone oxidoreductase
ND4L: NADH-ubiquinone oxidoreductase chain 4L
ATP6: ATP synthase membrane subunit 6
mtDNA: Mitochondrial DNA
gDNA: Genomic DNA
GEMs: Gel bead-in-emulsions
tRNA: Transfer RNA
NGS: Next generation sequencing
PCGs: Protein-coding genes
ML: Maximum likelihood
MRCA: Most recent common ancestor
CI: Credibility interval
BPP: Bayesian posterior probabilities
ESS: Effective sample size
CRH-U: Centre de Recherche en Hydrobiologie-Uvira
