# Supplementary material for: Complete mitochondrial genomes and updated divergence time of the two freshwater clupeids endemic to Lake Tanganyika (Africa) suggest intralacustrine speciation
Source: BMC Ecol Evol. 2022 Nov 3;22:127. doi: 10.1186/s12862-022-02085-8 (PMC9635120; doi:10.1186/s12862-022-02085-8)
Supplement: Supplementary file 2 — Additional file 2. Phylogenetic analysis and divergence time dating of taxon-reduced datasets focusing on Dorosomatinae. [file 12862_2022_2085_MOESM2_ESM.docx]

# Additional file 2: phylogenetic analysis and divergence time dating of taxon-reduced datasets focusing on Dorosomatinae

## Methods

Our original BEAST analyses for dating of divergence times only included the first and second codon positions of the 13 PCGs in order to achieve convergence. Since variable regions, such as the D-loop, regions coding for rRNA and third codon positions, can be informative for estimating recent divergence times, we repeated our BEAST analyses with two taxon-reduced datasets (see next paragraph) in favour of including more variable sites. For each dataset, two independent BEAST runs were conducted for 100 million generations before deciding non-convergence (ESS < 200 for one or more parameters). We also repeated analyses of phylogenetic content, maximum likelihood and Bayesian inference for these datasets, including all sites. See main text for detailed methods.

The first taxon-reduced dataset was focused on Dorosomatinae, including all 34 representatives of this subfamily and *Denticeps clupeoides* as the outgroup taxon. For calibration we chose with a single secondary calibration point from Bétancur et al. 2015 (MRCA of *D. clupeoides* and other clupeiforms at 188.9 MYA). The second taxon-reduced dataset also included all 34 representatives of Dorosomatinae, as well as five members of Alosinae, Clupeinae, Engraulidae and Coilinae each, and *D. clupeoides*, *Danio rerio*, *Carassius auratus* and *Cyprinus carpio* as the outgroup. This selection of taxa allowed the inclusion of all secondary calibration points outlined in the main text.

## Results & discussion

Phylogenetic content of the first taxon-reduced dataset (16614 bp) was higher (94.9% resolved quartets in TREE-PUZZLE) than that of the full dataset (88.5%). In the phylogenies resulting from this dataset, we did not observe any topological changes compared to the original ones (Figs. S1-S2). *Microthrissa* remained paraphyletic, with even better support in the new Bayesian tree (Figure S2). In the new phylogenies, *Sardinella lemuru* was also placed with other species of *Sardinella* with high confidence, which was not the case for the complete dataset. The sister relationship between *Ethmalosa fimbriata* and Pellonulini was not supported (Figs. S1-S2). Deeper nodes within Dorosomatinae also remained unresolved with similar support values as in the original phylogenies which most likely indicates a problem of insufficient taxon sampling. It does not seem to be related to substitution saturation at the 3rd codon position or hypervariable D-loop, as indicated by substitution saturation tests in DAMBE (Iss < Iss.c , P < 0.001). BEAST analyses did not converge for this dataset, even after sequentially omitting the D-loop and rRNA sequences.

The phylogenetic content of the second taxon-reduced dataset (17238 bp) was only slightly higher (90.9% resolved quartets in TREE-PUZZLE) than that of the complete dataset (88.5%). ML and Bayesian analyses did not produce different topologies than the complete dataset (Figs. S3-S4), but Bayesian analysis did provide higher resolution within Dorosomatinae, once more rendering *Microthrissa* paraphyletic with high support. In addition, *Gudusia chapra* was placed with species of *Tenualosa*, in accordance with Egan et al. (2018) and Bloom & Lovejoy (2014). In contrast to these studies, *Anodontostoma chacunda* was placed with *Nematalosa*, *Konosirus punctatus* and *Clupanodon thrissa*. *Hilsa kelee, Dorosoma, Sardinella* and *Harengula jaguana* also clustered together in this Bayesian phylogeny, and *Escualosa thoracata* and *Amblygaster sirm* formed a well-supported clade (Figure S4). BEAST analyses did also not converge for this dataset, even after sequentially omitting the D-loop and rRNA sequences.


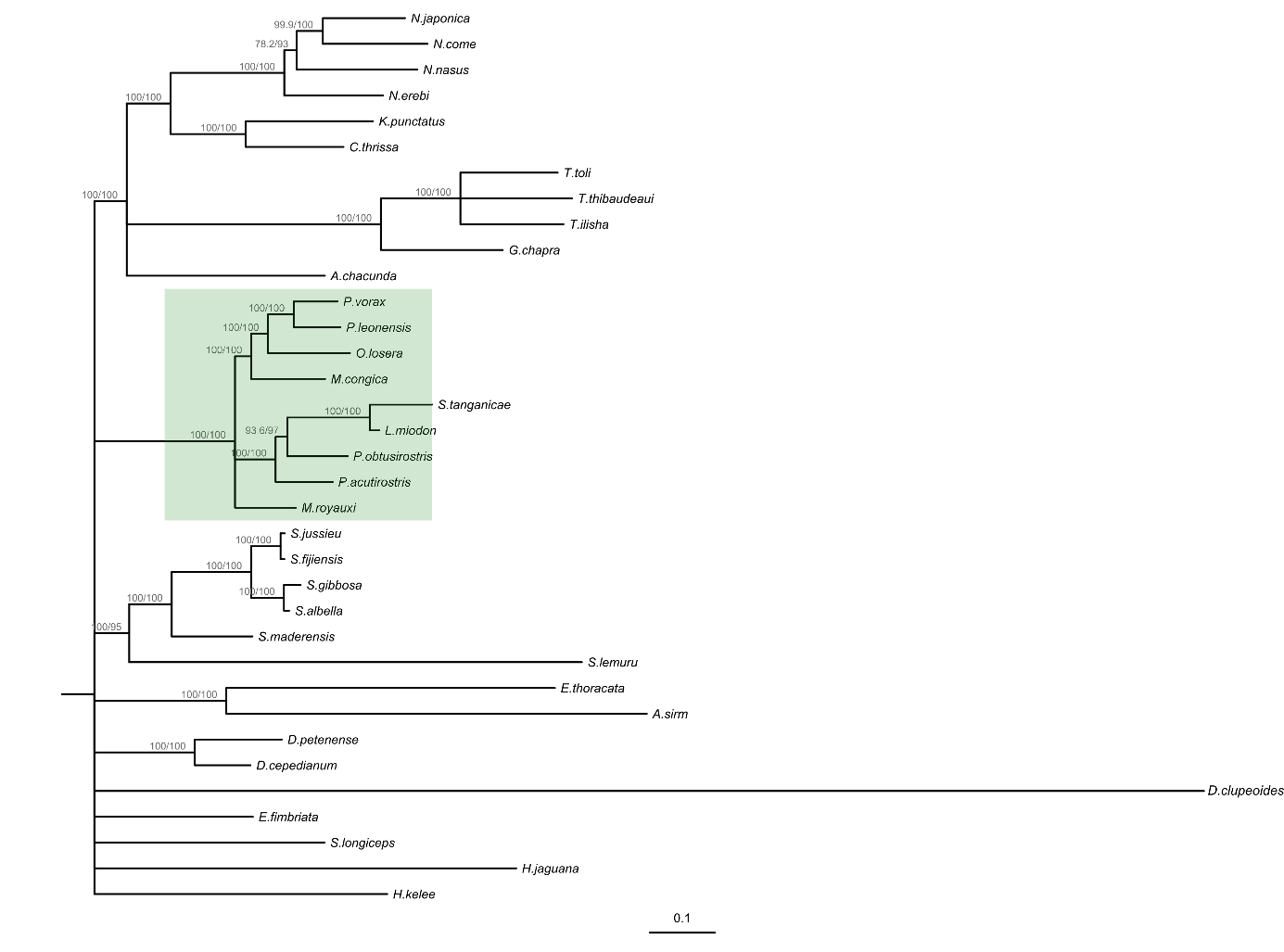


Figure S1. Outgroup-rooted maximum likelihood phylogeny of Dorosomatinae. Topology and branch lengths were estimated based on mitochondrial protein-coding genes, rRNA genes and D-loop sequence of 34 Dorosomatinae and *Denticeps clupeoides* (outgroup). Node support was assessed by Shimodaira-Hasegawa-like approximate likelihood ratio tests (SH-aLRT%) and ultrafast bootstrap (UFBoot%). Nodes with SH-aLRT% < 75 and UFBoot% < 90 were polytomized and their support values are not shown. The scale bar indicates model-corrected evolutionary distance (expected number of nucleotide substitutions per site). Pellonulini is highlighted in green.


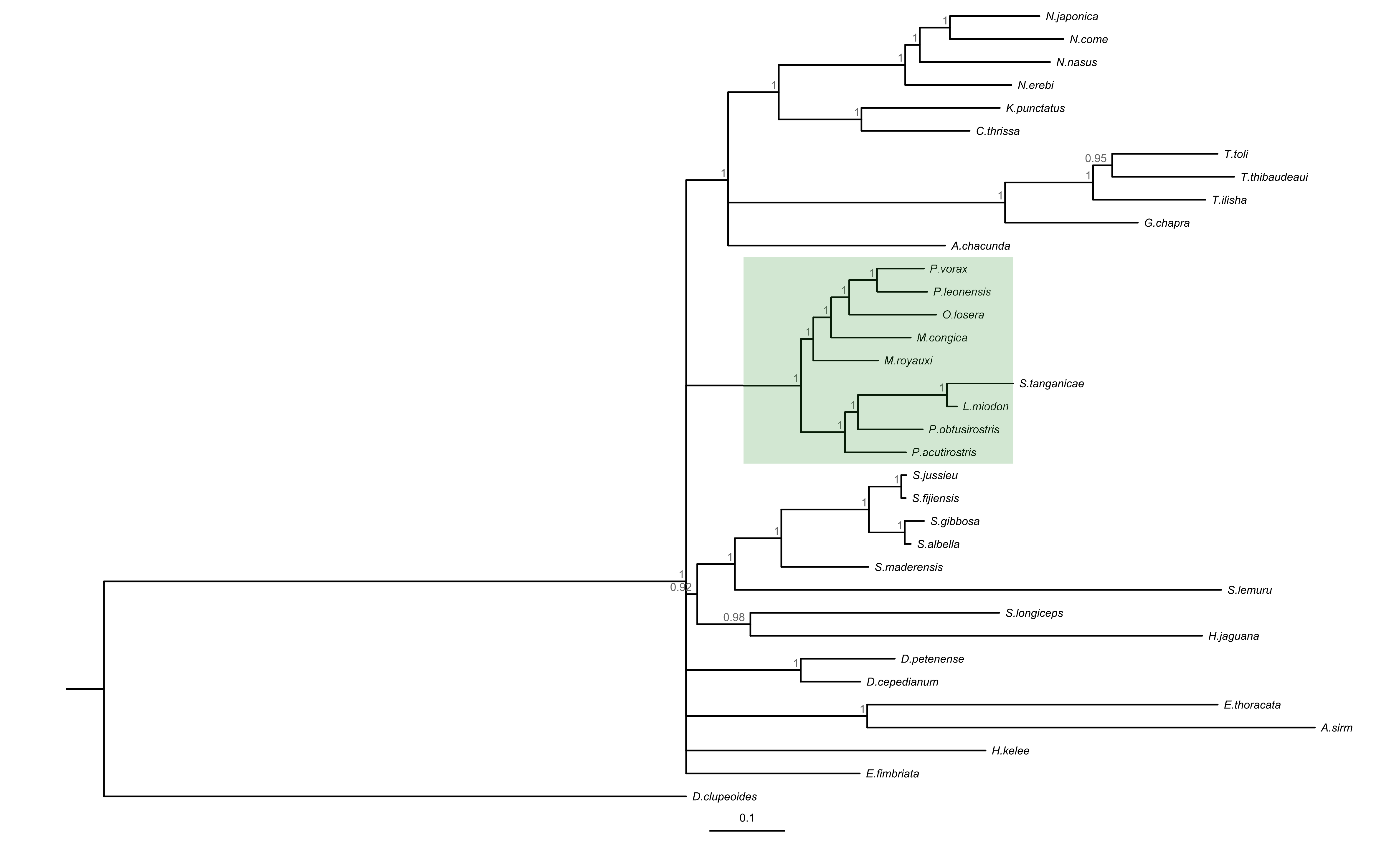


Figure S2. Outgroup-rooted Bayesian phylogeny of Clupeiformes. Topology and branch lengths were estimated based on mitochondrial protein-coding genes, rRNA genes and D-loop sequence of 34 Dorosomatinae and *Denticeps clupeoides* (outgroup). Node support was assessed by Bayesian posterior probabilities (BPP). Nodes with BPP < 0.85 were polytomized and their support values are not shown. Probabilities were rounded to the nearest 0.01. The scale bar indicates model-corrected evolutionary distance (expected number of nucleotide substitutions per site). Pellonulini is highlighted in green.


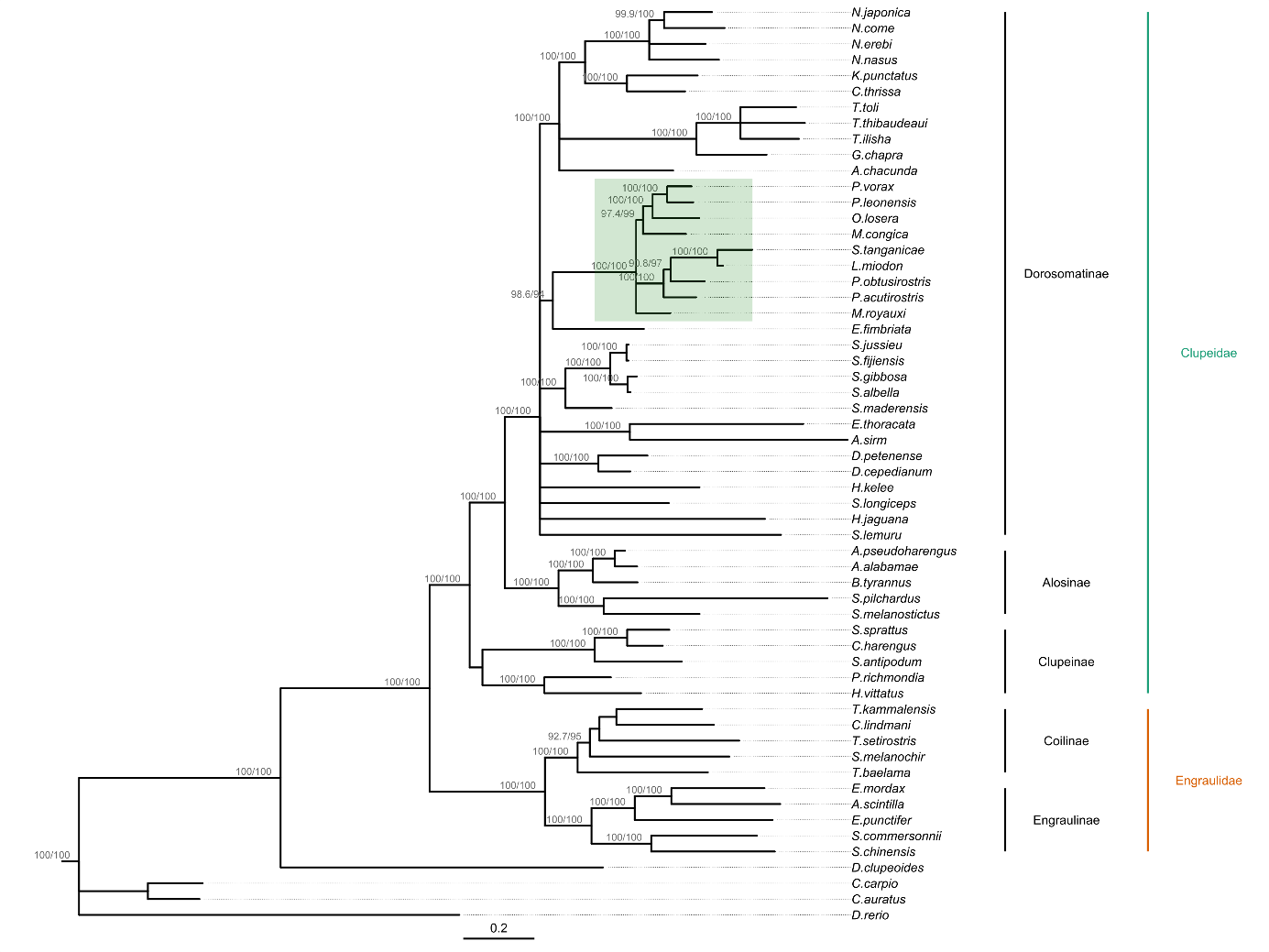


Figure S3. Outgroup-rooted maximum likelihood phylogeny of some subfamilies of Clupeiformes, focusing on Dorosomatinae. Topology and branch lengths were estimated based on mitochondrial protein-coding genes, rRNA genes and D-loop sequence of 54 clupeiforms and 4 non-clupeiforms. Node support was assessed by Shimodaira-Hasegawa-like approximate likelihood ratio tests (SH-aLRT%) and ultrafast bootstrap (UFBoot%). Nodes with SH-aLRT% < 75 and UFBoot% < 90 were polytomized and their support values are not shown. The scale bar indicates model-corrected evolutionary distance (expected number of nucleotide substitutions per site). Subfamilies are indicated on the right side in black, families in colour. Pellonulini is highlighted in green.


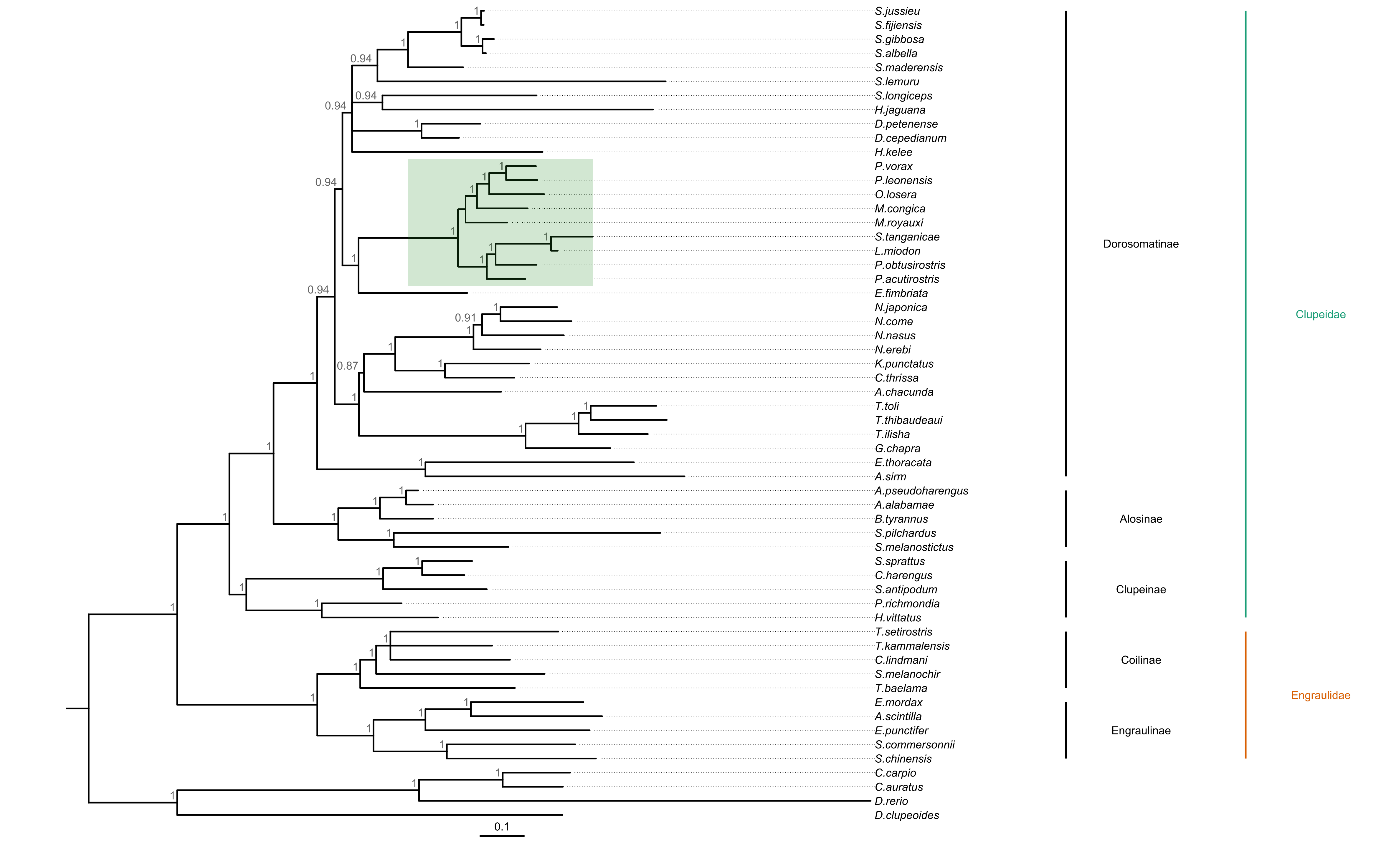


Figure S4. Outgroup-rooted Bayesian phylogeny of Clupeiformes. Topology and branch lengths were estimated based on mitochondrial protein-coding genes, rRNA genes and D-loop sequence of 54 clupeiforms and 4 non-clupeiforms. Node support was assessed by Bayesian posterior probabilities (BPP). Nodes with BPP < 0.85 were polytomized and their support values are not shown. Probabilities were rounded to the nearest 0.01. The scale bar indicates model-corrected evolutionary distance (expected number of nucleotide substitutions per site). Subfamilies are indicated on the right side in black, families in colour. Pellonulini is highlighted in green.
